# Data analysis on the three defect wavelengths of a MoS_2_-based defective photonic crystal using machine learning

**DOI:** 10.1038/s41598-023-49013-4

**Published:** 2023-12-07

**Authors:** Narges Ansari, Atieh Sohrabi, Kimia Mirbaghestan, Mahdieh Hashemi

**Affiliations:** 1 Department of Atomic and Molecular Physics, Faculty of Physics, Alzahra University, Tehran, 1993893973 Iran; 2 Department of Physics, College of Science, Fasa University, Fasa, 74617-81189 Iran

**Keywords:** Optical materials and structures, Nanoscale materials, Two-dimensional materials

## Abstract

To reduce the dimension of optoelectronic devices, recently, Molybdenum disulfide (MoS_2_) monolayers with direct bandgap in the visible range are widely used in designing a variety of photonic devices. In these applications, adjustability of the working wavelength and bandwidth with optimum absorption value plays an important role. This work proposes a symmetric defective photonic crystal with three defects containing MoS_2_ monolayer to achieve triple narrowband defect modes with wavelength adjustability throughout the Photonic Band Gap (PBG) region, 560 to 680 nm. Within one of our designs remarkable FWHM approximately equal to 5 nm with absorption values higher than 90% for the first and third defect modes are achieved. The impacts of varying structural parameters on absorption value and wavelength of defect modes are investigated. Due to the multiplicity of structural parameters which results in data plurality, the optical properties of the structure are also predicted by machine learning techniques to assort the achieved data. Multiple Linear Regression (MLR) modeling is used to predict the absorption and wavelength of defect modes for four datasets based on various permutations of structural variables. The machine learning modeling results are highly accurate due to the obtained R^2^-score and cross-validation score values higher than 90%.

## Introduction

In recent years, two-dimensional materials, the most important of which are graphene and Transition Metal Dichalcogenides (TMDCs), have been applied widely in optoelectronic applications^1–3^. Graphene monolayer among all 2D materials made extensive use in optical devices because of low loss, and intense light absorption^4,5^. However, TMDCs with a thickness-dependent bandgap, in contrast to the zero bandgap of graphene, are very good candidates for use in electronic devices such as field-effect transistors, optical sensors, memories, and solar cells^6,7^.

One of the special properties of TMDCs is their indirect bandgap in the bulk state, which can be transformed into a direct bandgap by reducing the number of layers to a single monolayer^8–10^. The reduction of TMDC layers to one, results in high absorption in the visible-light range in these nanometric materials^11^. Generally, TMDCs are represented as MX_2_, where M denotes the transition metals like Molybdenum (Mo), Tungsten (W), … and X represents the chalcogen, such as Sulfur (S), Selenium (Se), …^12,13^.

One of the most studied TMDCs is the Molybdenum disulfide (MoS_2_) monolayer, composed of three-layer sheets of three-dimensionally bonded sulfur and molybdenum^14,15^. These layers are weakly bonded to each other through van der Waals forces, which makes the MoS_2_ monolayer a reactive nanometric material due to the presence of free electrons^9,16,17^. The MoS_2_ monolayer provides remarkable absorption peaks of 23%, 6%, and 7% due to its direct band gap at the wavelengths of 432 nm, 617 nm, and 664 nm, respectively^18^. Although the MoS_2_ monolayer is a noteworthy material due to the mentioned properties, its absorption value must be increased for optical and optoelectronic applications^19,20^.

There are different methods implemented by researchers to increase MoS_2_ absorption^21–23^. Depending on the need for high absorption in a broad or narrow bandwidth, the MoS_2_ monolayer can be used in different structures. For example, an atomic crystal structure consisting of MoS_2_ monolayer, modeled as non-Hermitian photonic scattering with an absorption value over 50% in a broadband spectral range over 100 nm, is suggested in^24^. Using the MoS_2_ monolayer as the repeating layer in one- or two-dimensional Photonic Crystals (PCs) and Quasi-PCs (QPCs) is suggested in Ref.^25–27^ to achieve high absorption in a wide bandwidth. These structures forbid the light to propagate in a range of frequencies, which is called the Photonic Band Gap (PBG) and is highly sensitive to structural conditions^28^. In another work, it is reported that the bandwidth of absorption peaks can be tuned by plasmonic structures consisting of gold gratings on MoS_2_ monolayers^29^.

Since the MoS_2_-based narrowband absorbers play an important role in sensing applications, researchers suggested different structures based on the MoS_2_ monolayer to reach this goal^30–32^. As reported in Ref.^33^, by inserting a MoS_2_ monolayer as a single defect in a PC or QPC, a Defective PC (DPC) or Defective QPC (DQPC) will be constructed which provides an absorption peak above 90%. In such structures, the defect makes waves pass through a narrow range of wavelengths and provides high absorption in the PBG, known as the defect mode^34,35^. The number of defects in a DPC and the symmetry or asymmetry of the structure concerning the defects are two of the most important factors affecting the number of defect modes^36^. For example, a symmetric DPC with two defects provides two defect modes in the PBG, while an asymmetric one provides four defect modes on the edge of the PBG^37^. Another research showed that DPCs containing two defects are good candidates for fabrication of ultrafast all-optical switching devices^38^. In another design, a PC with triple-defect applicable in polarization control is proposed recently^39^. The defect modes’ absorption value and peak wavelength depend on the distance between the defects, the incident light angle, and the thickness of the defect layers^40^.

Although increasing the number of defects in a DPC modifies the absorption value, wavelength, and Full Width at Half Maximum (FWHM) of defect modes, it complicates the design because of structural parameters’ abundance that leads researchers to use machine learning techniques. In the field of PCs, machine learning is being used to design and optimize a wide range of devices and structures, such as optical waveguides, resonant cavities, and optical sensors^41–43^. To design DPC structures and predict their properties, various machine learning methods such as linear, polynomial, and (KNN) regression are implemented through training a model on a training subset and evaluating its validity on a test subset to improve its generalization ability^44–47^.

In this work, a symmetric DPC structure with three defects based on MoS_2_ monolayer that provides three defect modes in the PBG region is proposed. As we have three defects in our structure and variety of tuning parameters, the wavelength of the defect modes can be adjusted precisely, while we show that we could keep the absorption values high enough. Due to the data abundance in our DPC which arises from different defect’s displacements, using a machine learning technique is crucial.

After examining different machine learning methods, we concluded that a model based on Multiple Linear Regression (MLR) is most accurate to predict absorption and wavelength of the defect modes and is applied to assort the achieved results. Formulation of wavelength and absorption of the defect modes according to the MLR models with cross-validation score above 90% is reached which can be implemented to predict the functionality of our proposed DPC with high accuracy. Python 3.10 is used for both simulating the proposed DPC structure and machine learning of its functionality. The method used in this paper can be generalized in other DPC structures and predict their optical properties by applying proper machine learning model.

## Design and results

### Structure

**Figure 1:**
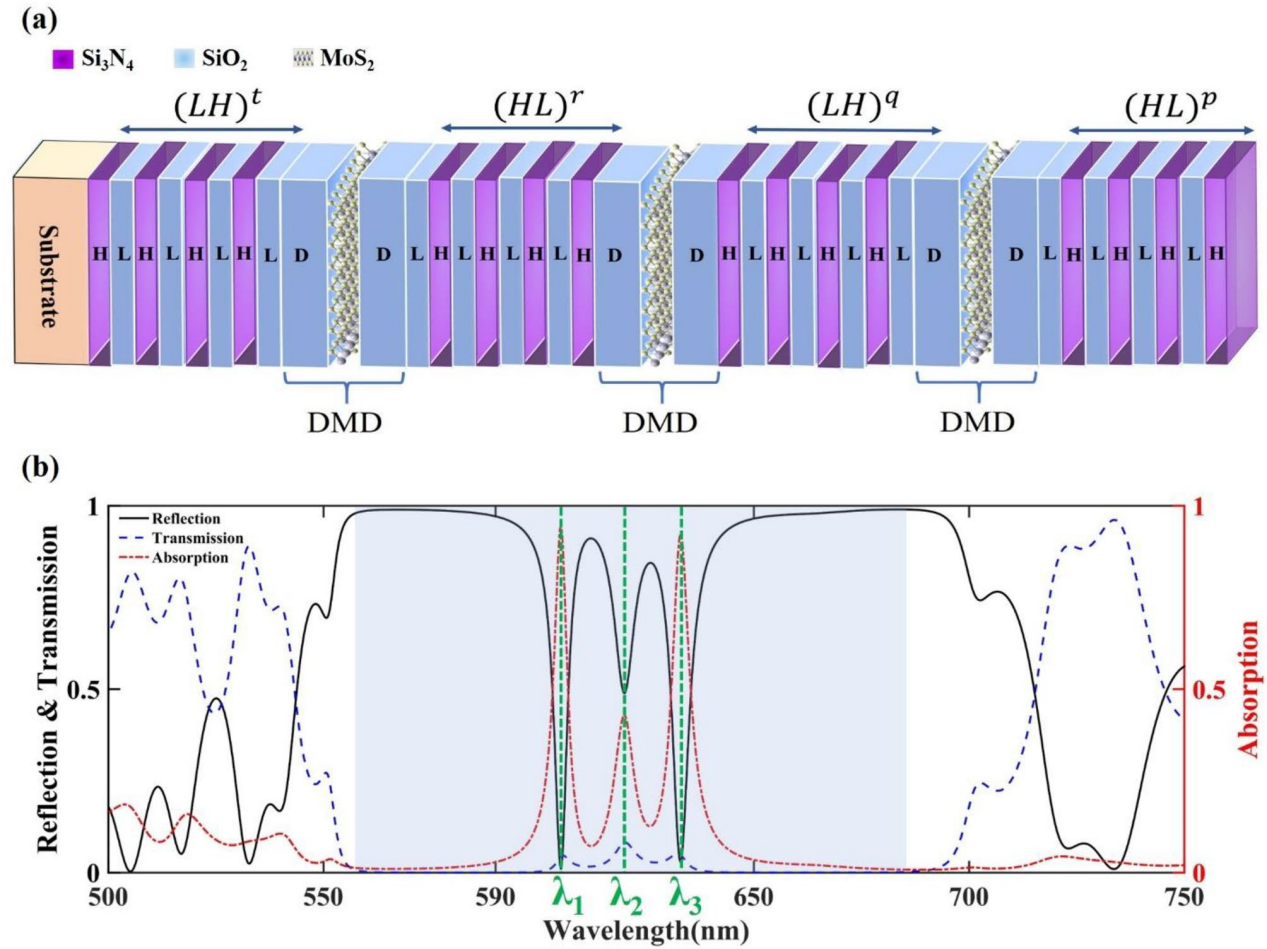
(**a**) Schematic of ${(HL)}^{p} DMD {(LH)}^{q} DMD {(HL)}^{r} DMD {(LH)}^{t} /substrate$. The p, q, r, and t parameters demonstrate the number of $HL$ or $LH$ layers repetition which their changes are studied throughout the paper. (**b**) The Absorption, transmission, and Reflection spectra for p = 4, q = 4, r = 7, and t = 8. The shaded region illustrates the PBG with three defect modes at λ_1_, λ_2_, and λ_3_.

To reach high absorption in narrowband wavelengths, we introduce a symmetric DPC, ${(HL)}^{p} DMD {(LH)}^{q} DMD {(HL)}^{r} DMD {(LH)}^{t} /substrate$, as schematically represented in Fig. 1a. Symmetry of the DPC means that the layers closest to the defects are the same on both sides. This structure is composed of consecutive periodic structures including a higher refractive index layer, denoted by H, which we set to be Si_3_N_4_, and a lower refractive index layer which we choose to be SiO_2_ and is shown by L, all layers are placed on a SiO_2_ substrate.

These periodic structures are separated by three similar defects, which are depicted as DMD. The number of periodicities of these periodic structures is shown by *p* (the number of periodicities on the top of the first defect), *q* (the number of periodicities between the first and second defect), *r* (the number of periodicities between the second and third defect), and *t* (the number of periodicities between third defect and substrate) parameters. The defects are designed as DMD in which D and M are chosen to be SiO_2_ and the monolayer of MoS_2_. To localize the incident light in the defect layer, the M monolayer is sandwiched between two D layers to make the defect thickness the same order as the incident light wavelength.

To simulate the optical properties of our introduced structure, the Transfer Matrix Method (TMM) is used^48^. The incident light illuminates the structure normally. The transfer matrix of each layer for a normal incident can be expressed as:

1 $${M}_{j}=\left(\begin{array}{cc}{\text{cos}}\left(\frac{2\pi {n}_{j}{d}_{j}}{\lambda }\right)& -i \frac{1}{{n}_{j}}{\text{sin}}\left(\frac{2\pi {n}_{j}{d}_{j}}{\lambda }\right)\\ -i {n}_{j}{\text{sin}}\left(\frac{2\pi {n}_{j}{d}_{j}}{\lambda }\right)& {\text{cos}}\left(\frac{2\pi {n}_{j}{d}_{j}}{\lambda }\right)\end{array}\right)$$

in which ${n}_{j}$ and ${d}_{j}$ denote the refractive index and thickness of the ${j}^{th}$ layer, and λ represents the wavelength of the incident light. The ultimate transfer matrix M for the entire structure can be obtained by multiplying each constituent layer’s transfer matrix. The tangential electric and magnetic field for the initial $({E}_{0t},{H}_{0t})$ and last $({E}_{st},{H}_{st})$ surrounding layers can be given by the following equation:

2 $$\left(\begin{array}{c}{E}_{st}(r)\\ {H}_{st}(r)\end{array}\right)=\left(M\right)\left(\begin{array}{c}{E}_{0t}\left(r\right)\\ {H}_{0t}\left(r\right)\end{array}\right),$$

Here 0 and s refer to air and substrate. Additionally, amplitudes of transmitted (${c}^{+}$) and reflected (${c}^{-}$) fields in each layer are

3 $$\left(\begin{array}{c}{E}_{0t}\\ {H}_{0t}\end{array}\right)=\left(\begin{array}{cc} {e}^{i\frac{2r\pi {n}_{0}}{{\lambda }_{0}}}& {e}^{-i\frac{2r\pi {n}_{0}}{{\lambda }_{0}}}\\ {{n}_{0}e}^{i\frac{2r\pi {n}_{0}}{{\lambda }_{0}}}& -{n}_{0}{e}^{-i\frac{2r\pi {n}_{0}}{{\lambda }_{0}}}\end{array}\right)\left(\begin{array}{c}{c}_{0}^{+}\\ {c}_{0}^{-}\end{array}\right),$$

4 $$\left(\begin{array}{c}{E}_{st}\\ {H}_{st}\end{array}\right)=\left(\begin{array}{cc} {e}^{i2r\pi {n}_{s}\frac{{\text{cos}}{\theta }_{s}}{{\lambda }_{s}}}& {e}^{-i2r\pi {n}_{s}\frac{{\text{cos}}{\theta }_{s}}{{\lambda }_{s}}}\\ {{n}_{s}e}^{i2r\pi {n}_{s}\frac{{\text{cos}}{\theta }_{s}}{{\lambda }_{s}}}& -{n}_{s}{e}^{-i2r\pi {n}_{s}\frac{{\text{cos}}{\theta }_{s}}{{\lambda }_{s}}}\end{array}\right)\left(\begin{array}{c}{c}_{s}^{+}\\ 0\end{array}\right).$$

Finally, transmission, reflection, and absorption are determined, $T=\frac{{n}_{s}}{{n}_{0}}{\left|\frac{{c}_{s}^{+}}{{c}_{s}^{-}}\right|}^{2}$, $R={\left|\frac{{c}_{0}^{+}}{{c}_{0}^{-}}\right|}^{2}$, and $A=1-T-R$. In the TMM method, the refractive index and thickness of each layer are required. The refractive indices of SiO_2_ and Si_3_N_4_ are obtained from ${n}_{Si{O}_{2}}=\sqrt{1.288604141+\frac{1.07044083{\lambda }^{2}}{{\left(\lambda \times {10}^{-3}\right)}^{2}-1.00585997\times {10}^{-2}}+\frac{1.10202242{\lambda }^{2}}{{\left(\lambda \times {10}^{-3}\right)}^{2}-100}}$ and ${n}_{S{i}_{3}{N}_{4}}=\sqrt{1+\frac{2.8939{\left(\lambda \times {10}^{-3}\right)}^{2}}{{\left(\lambda \times {10}^{-3}\right)}^{2}-{0.13967}^{2}}}$, respectively^49,50^. The complex refractive index of the MoS_2_ monolayer is ${n}_{{MoS}_{2}}=n+ik=\sqrt{{\varepsilon }_{{MoS}_{2}}}$, here, ${\varepsilon }_{{MoS}_{2}}$, *n*, and *k*, show permittivity, refractive, and extinction coefficients, respectively, and could be obtained from the Lorentz equation

5 $${\varepsilon }_{{MoS}_{2}}=\varepsilon \left(\infty \right)+\sum_{\beta =1}^{N}\frac{{A}_{\beta }}{{\omega }_{\beta }^{2}-{\omega }^{2}-i\omega {B}_{\beta }}.$$

According to Eq. (5), *ω* and ${\varepsilon }_{\infty }$ are incident light frequency and the DC permittivity is about 2.2. ${\omega }_{\beta }$, ${A}_{\beta }$, and ${B}_{\beta }$ are the resonant frequency, the oscillation power, and the damping factor of the ${\beta }^{th}$ oscillator, and their values are taken from^51^.

The thickness of constituent layers is taken from ${d}_{L}=\frac{{\lambda }_{des}}{4{{(n}_{L})}_{{\lambda }_{des}}}$,${d}_{H}=\frac{{\lambda }_{des}}{4{{(n}_{H})}_{{\lambda }_{des}}}$, and ${d}_{D}=\frac{{\lambda }_{des}}{2{{(n}_{D})}_{{\lambda }_{des}}}$, in which ${\lambda }_{des}$ is the design wavelength and it is chosen as 617 nm. ${{(n}_{L})}_{{\lambda }_{des}}$, ${{(n}_{H})}_{{\lambda }_{des}}$, and ${{(n}_{D})}_{{\lambda }_{des}}$ represents the refractive indices of L, H, and D layers in the design wavelength. By performing these calculations, ${d}_{L}$, ${d}_{H}$, and ${d}_{D}$ are obtained as 99.9 nm, 76.6 nm, and 199.9 nm, respectively. The thickness of the MoS_2_ monolayer (${d}_{M}$) is set to be 0.6 nm based on experimental research^11^.

Insertion of a defect layer in PCs causes excitation of a narrow band absorption peak in the PBG. Increasing the number of included defect layers affects the number of defect modes. Among the studied structures, the absorption, transmission, and reflection spectra of structure with *p* = 4, *q* = 4, *r* = 7, and *t* = 8, are demonstrated in Fig. 1b. The shaded region, which is widened between 560 and 680 nm, illustrates the PBG of the structure with nearly zero transmission except at the wavelengths of the excitation of the three defect modes (λ_1_, λ_2_, and λ_3_). The three defect modes wavelengths are located at 606 nm, 621 nm, and 634 nm with absorption values of 0.94, 0.42, and 0.92 (A_1_, A_2_, and A_3_), respectively.

### Analysis of the structure

The presence of the MoS_2_ monolayer as the only material with a complex refractive index, causes light absorption in the structure. The localization of the light in the wavelength of defect modes in the DMD, causes successive reflections from these layers. This phenomenon causes constructive and destructive interference of reflected waves, which creates absorption peaks in the PBG region. The constructive or destructive interferences of these waves depend on the DMD’s location in the structure. Therefore, changing the structural parameters affects the wavelength and absorption values of the defect modes.

**Figure 2:**
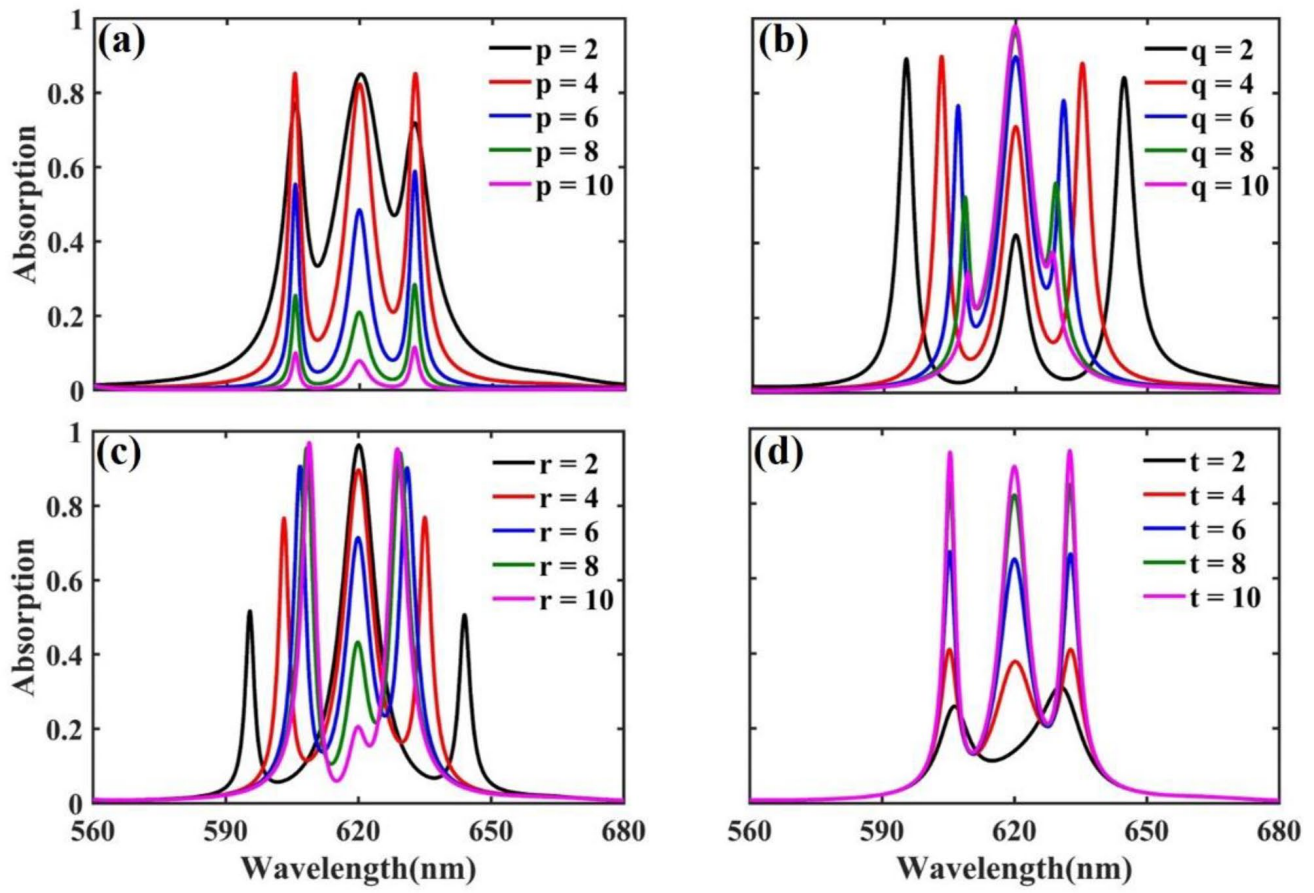
Absorption spectra in the PBG as a function of wavelength by (**a**) different values of p and keeping constant q = 4, r = 6, and t = 8, (**b**) different values of q with constant parameters of p = 4, r = 6, and t = 8, (**c**) different values of r with p = 4, q = 4, and t = 8, and (**d**) different values of t by fixing values of p = 4, q = 4, and r = 6.

To investigate the effects of changing *p*, *q*, *r*, and *t* parameters on the absorption spectra of the introduced structure, the absorption spectra in the PBG for different values of these parameters are demonstrated in Fig. 2. The effect of changing the *p* parameter on absorption spectra for *q* = 4, *r* = 6, and *t* = 8, is shown in Fig. 2a. It illustrates that the absorption of defect modes rises by increasing the *p* parameter up to* p* = 4 which has the most absorption peaks. Then, for *p* values greater than 4, it reduces, while their wavelengths, λ_1_, λ_2_, and λ_3_, are constant with different values of *p*. On the other hand, changing *p* affects the depth of the valley which is defined as ${D}_{valley}=({A}_{peak}-{A}_{valley})/{A}_{peak}$, where ${A}_{peak}$ is the most value of absorption between A_1_, A_2_, and A_3_, and ${A}_{valley}$ stands for the lowest value of the absorption between λ_1_ and λ_3_. Whatever ${D}_{valley}$ tends to 1, the defect modes are more distinguishable, and the nearest value of ${D}_{valley}$ to 1, occurs with *p* = 4 in our studied case. Due to the higher value of both absorption and ${D}_{valley}$ of the structure with *p* = 4, this value is selected for further studies.

The absorption spectra for *q* values varying from 2 to 10 by steps of 2 for *p* = 4, *r* = 6, and *t* = 8, are plotted in Fig. 2b. As it can be seen, although changing *p* keeps the defect mode wavelengths constant, varying *q* adjusts them with values of λ_1_ and λ_3_ while λ_2_ remains constant. The increment of *q* leads to a redshift of λ_1_ and a blueshift of λ_3_, which leads these two defect mode wavelengths to approach the middle one, λ_2_. It also can be deduced from Fig. 2b that increasing *q* reduces A_1_ and A_3_ while increasing A_2_.

By selecting the optimum value of 4 for *p* and choosing the *q* value of 4 in Fig. 2c, the effect of changing *r* on absorption spectra is studied by considering *t* = 8. Similar to the *q* change, by *r* increment, λ_1_ and λ_3_ are approaching λ_2_, while λ_2_ is constant. But in the context of the absorption value, in contrast to the *q* change, increasing the *r* parameter increases A_1_ and A_3_ while causing a reduction in A_2_.

In Fig. 2d, the absorption spectra are plotted for five consecutive even values of the *t* parameter beginning from 2, where *p* = *q* = 4 and *r* = 6. Like the effect of changing the *p* parameter, different values of *t* would not affect the wavelength of defect modes, while, increasing *t* increases the absorption of all three defect modes up to *t* = 8. More increases in the *t* value, would not make an impressive change in the absorption value of the defect modes. Therefore, *t* = 8 is chosen as the optimum value in the structure due to the advantage of using the smaller total number of layers in experimental works.

**Figure 3:**
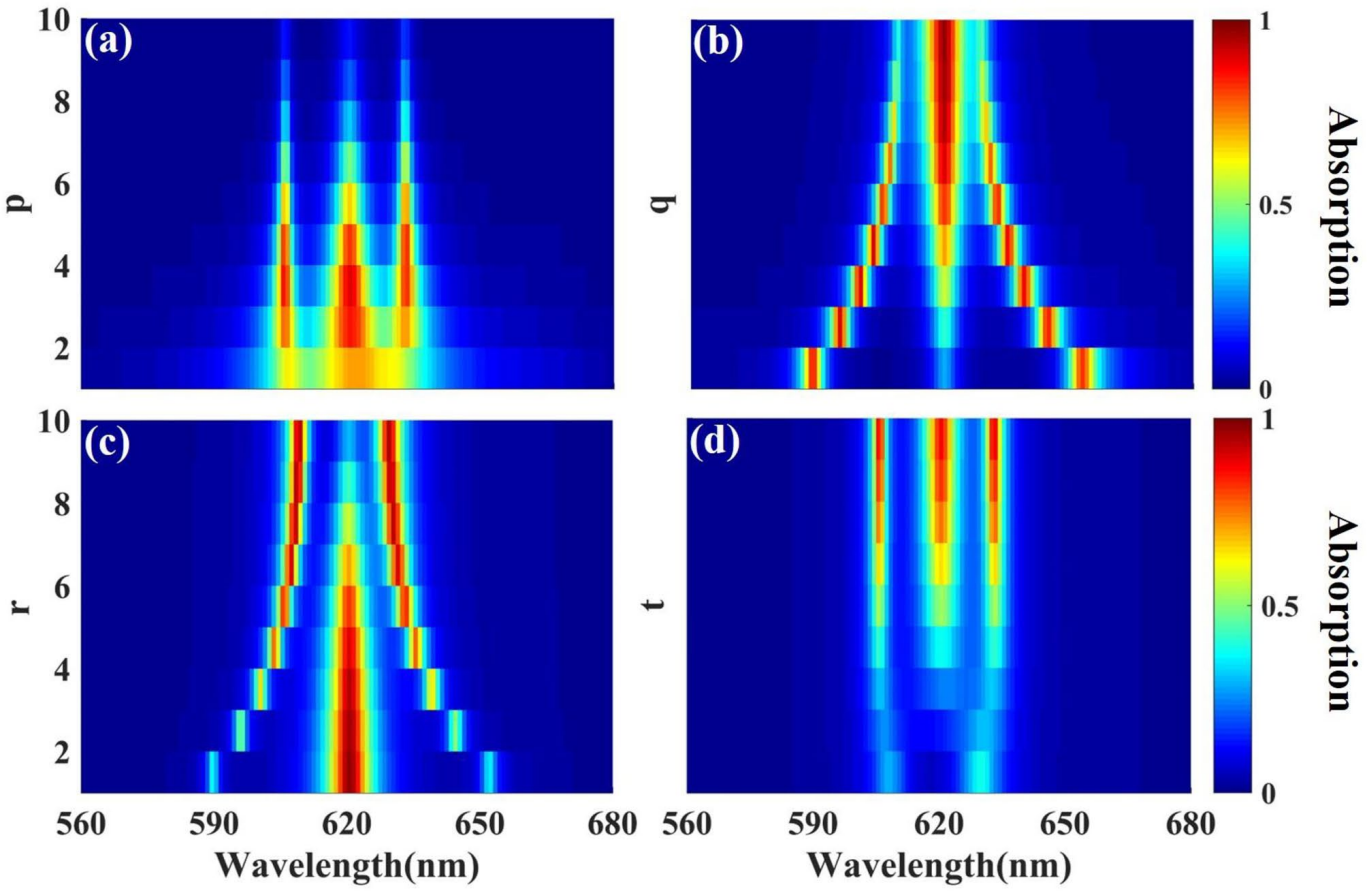
Absorption spectra of defect modes by varying wavelength and (**a**) p, (**b**) q, (**c**) r, and (**d**) t. All constant parameters in each part of the figure are selected as mentioned in corresponding part of Fig. 2. The color bar is demonstrative of absorption value.

To clarify the effect of changing *p*, *q*, *r*, and *t* parameters on the wavelength of defect modes and their FWHM, the absorption spectra for different values of *p* (for fixed values of *q* = 4, *r* = 6, and *t* = 8), *q* (in case of *p* = 4,* r* = 6, and *t* = 8), *r* (with *p* = 4, *q* = 4, and *t* = 8), and *t* (taking *p* = 4,* q* = 4, and *r* = 6) are demonstrated in Fig. 3a–d, respectively. According to Fig. 3a,d, it can be observed that changing *p* and *t* parameters will not make any impressive changes on defect mode wavelengths while considering Fig. 3b,c, q and r parameters would affect first and third defect mode wavelengths. The second defect mode is located at 621 nm, in the middle of the PBG, which changing any parameters, *p*, *q*, *r*, or *t*, will not affect its wavelength. In symmetric DPC structures with one defect, a single defect mode with minimum wavelength changes concerning the structural parameters is located in the middle of the PBG, near the design wavelength^52^. By increasing the number of defects with the condition that all of them are symmetrical, the defect mode located in the middle of PBG remains constant and other modes are added around it.

Increasing *q* compared with *r* has inverse effects on the absorption value of defect modes. In a way, increasing *q* leads to an A_2_ increment and decrement of A_1_ and A_3_, contrariwise, increasing *r* results in a decrement of A_2_ and increment of A_1_ and A_3_. This way, tuning of the defect mode wavelengths is possible by changing *q* and *r* parameters, while, the amount of absorption can be adjusted by varying any of *p*, *q*, *r*, and t parameters.

Careful investigation of Fig. 3a illustrates that by increasing the *p* parameter more than 8, the absorption value reduces until it becomes zero for all three defect modes. Therefore, all three defect modes will eventually disappear, and the structure will act like a perfect PC. Figure 3b shows that, while, for *q* values less than 10 three different defect modes can be recognized, increasing *q* to the values greater than 10 reduces the defect modes to one. This phenomenon occurs as the incoming light can’t reach the second defect of the structure and behaves like a DPC with one symmetric defect^52^. Such a happening with *r* values greater than 10 can be also deduced from Fig. 3c, in which a reduction of the number of the defect modes from three to two occurs. The appearance of these two defect modes is a characteristic behavior of a DPC with two symmetric defects^37^. In the case of Fig. 3d, as the parameter *t* decreases, the middle defect mode gradually disappears and the two side defect modes converge, so that for *t* parameter less than 3, there are only two defect modes. According to the description of Fig. 3, to have three defect modes, the parameters *p*, *q*, and *r* must be less than or equal to 12, 15, and 12, respectively, and parameter *t* must be greater than or equal to 3. Within these limitations, the introduced DPC structure has three defect modes with different absorption values.

In addition to the value of absorption and wavelength, the FWHM of the defect modes can also be controlled by changing the so-called parameters. From the wavelength range with a high value of the absorption (the red color tone), it can be seen that the second defect mode’s FWHM can be affected more than the others by changing *p*, *q*, and *r* parameters.

**Figure 4:**
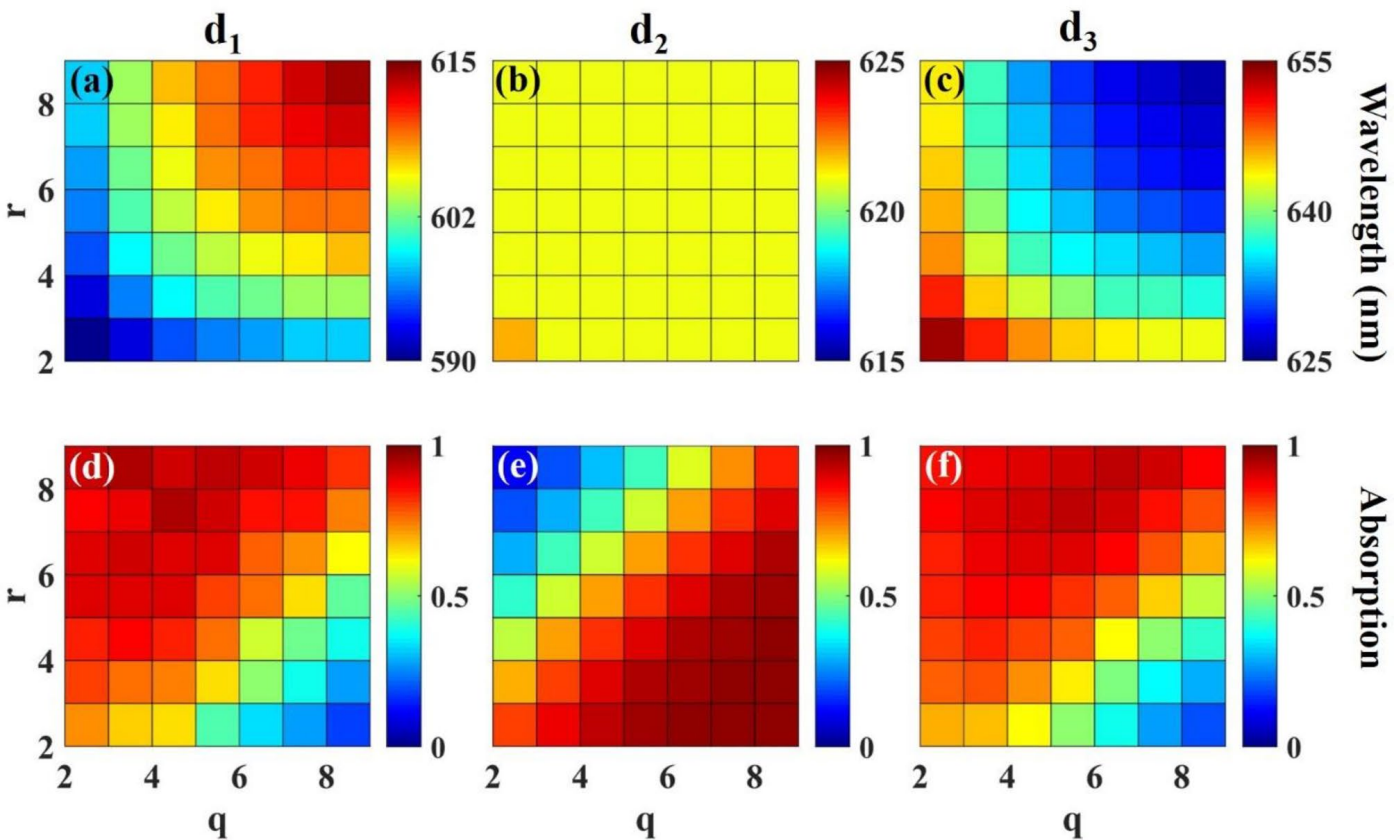
Wavelength/absorption of defect modes for d_1_ (**a**,**d**), d_2_ (**b**,**e**), and d_3_ (**c**,**f**) as a function of q and r while p = 4 and t = 8. The first row’s color bar demonstrates wavelength and the color bar of the second row represents the absorption value.

Considering the effective parameters, *q,* and *r*, on the first and third defect modes wavelength, in Fig. 4 we investigate the effect of simultaneous change of these two parameters on each defect mode absorption value and wavelength, separately. Optimum values of *p* = 4 and *t* = 8 are set in obtaining Fig. 4. We focus on the wavelength change of each defect mode, the first defect mode (d_1_), the second defect mode (d_2_), and the third defect mode (d_3_), respectively, in the first, second, and third columns of the first row of Fig. 4 with changing *q* and *r*. Their absorption behavior is discussed in the second row of this figure.

According to Fig. 4a,c, the first and third defect mode wavelengths can be tuned by changing *q* and *r* values. For both increasing *q* and *r*, the wavelength adjustability is redshift and blueshift for λ_1_ and λ_3_, respectively. Opposite to λ_1_ and λ_3_, based on Fig. 4b, there is no wavelength controlling for the middle defect mode, λ_2_. Considering the represented Absorption values of Fig. 4d,f, absorption values higher than 90% for both d_1_ and d_3_ with *q* and *r* parameters greater than 3 and less than 8, are achieved. While the absorption of d_2_ is nearly perfect for *q* and *r* values greater than 3, as shown in Fig. 4e. Selecting *q* and *r* values between 3 and 8 allows us to control the defect mode wavelengths, λ_1,_ and λ_3_, with acceptable absorption values.

To have an exact numerical view of the three investigated defect mode wavelengths, absorption, and FWHM, in Table 1, these values are reported for two cases of p = 4, 5 with q = 2, 4, 6, 8, r = 4, 8, and taking t = 8 as a constant. These values are extracted from Figs. 1, 3, and 4. Considering the included data, consistent with the discussed results, λ_2_ is constant for all different values of *p*, *q*, and *r* parameters while, by increasing *q* and *r*, λ_1_ has a red shift, whereas λ_3_ has a blue shift. Comparing the absorption of every three defect modes for *p* = 4 and *p* = 5 shows that the best value for *p* is 4.

**Table 1:** The value of absorption (A_1_, A_2_, and A_3_), wavelength (λ_1_, λ_2_, and λ_3_), and FWHM (FWHM_1_, FWHM_2_, and FWHM_3_) for each defect mode (d_1_, d_2_, d_3_) with different values of p, q, and r in constant value of t = 8.

Parameters			d_1_			d_2_			d_3_		
*p*	*q*	*r*	A_1_	λ_1_ (nm)	FWHM_1_ (nm)	A_2_	λ_2_ (nm)	FWHM_2_ (nm)	A_3_	λ_3_ (nm)	FWHM_3_ (nm)
4	2	4	0.83	595	4.1	0.56	621	6.2	0.80	647	5.2
4	4	4	0.84	602	3.3	0.82	621	6.8	0.80	638	3.7
4	6	4	0.58	605	2.6	0.94	621	8.4	0.62	635	2.8
4	8	4	0.38	607	1.8	0.97	621	8.9	0.41	633	1.9
4	2	8	0.92	598	4.3	0.12	621	4.4	0.85	644	5.7
4	4	8	0.92	607	4.1	0.30	621	3.8	0.90	633	4.9
4	6	8	0.92	611	3.1	0.58	621	3.0	0.93	628	5.4
4	8	8	0.82	614	2.2	0.83	621	2.2	0.87	626	11.1
5	2	4	0.87	595	3.1	0.39	621	5.7	0.87	647	3.8
5	4	4	0.71	602	2.6	0.66	621	5.7	0.74	638	3.3
5	6	4	0.41	606	2.5	0.84	621	6.7	0.45	635	2.7
5	8	4	0.25	607	1.8	0.93	621	6.9	0.28	633	1.8
5	2	8	0.97	598	3.3	0.07	621	4.3	0.96	643	4.1
5	4	8	0.92	607	3.0	0.19	621	3.7	0.94	633	3.9
5	6	8	0.83	611	2.6	0.41	621	2.9	0.89	628	4.0
5	8	8	0.66	614	2.2	0.68	621	2.2	0.75	626	9.7

To sort out the achieved wavelength and absorption data of the defect modes by considering their dependency on the parametric values of the structure, *p*, *q*, and *r*, and predicting their values for unstudied cases, machine learning of our data is unavoidable. In our data analysis, reminding the independence of the defect mode wavelengths to the *t* value, this parameter will not be included in our modeling and a fixed value of *t* = 8 is selected due to the inclusion of minimum repetition of layers with optimum absorption.

## Machine learning modeling results

To model the wavelength and absorption value of the defect modes, the division of our data set to 80% train and 20% test, is done in the Python scikit learn library. We will show that MLR can cover the wavelength and absorption of the defect mode’s dependency on the different geometrical parameters of the structure with minimum error. The MLR method aims to model the relationship between two or more independent variables and a dependent variable by fitting a linear equation to the training dataset and testing its validity by examining of the model on the test dataset. An MLR can be written theoretically as:

6 $$Y= {\theta }_{0}+{\theta }_{1}{X}_{1}+{\theta }_{2}{X}_{2}+\dots +{\theta }_{n}{X}_{n},$$

where *Y* is the dependent, and the ${X}_{i}$ s are the independent variables (*i* = 1, 2,…, *n*), with ${X}_{i}$ s also called regressors. The ${\theta }_{0}$ is the value of *Y* when all ${X}_{i}$ s are equal to 0 which is called the intercept. The ${\theta }_{i}$ s for *i* = 1, 2,…, *n* are the regression coefficients and finding them is the goal of applying the MLR. To find the model that best fits all available data, machine learning of them is performed based on dividing the data into two non-overlapping subsets. The first subset selects 80% of all data randomly and is used to train the model that fits well the “train data” and leads to finding the ${\theta }_{i}$ s. The remaining 20% of data which is named “test data”, is used to examine the precision efficiency of the model by checking the proximity of the predicted and actual data of the test part. The evaluation standard parameters that are commonly used to report the model performance include R^2^-score, Mean Squared Error (MSE), Root Mean Squared Error (RMSE), and Mean Absolute Error (MAE). The R^2^-score evaluates the performance of the model by measuring the squared correlation between the actual and predicted values. If ${\widehat{y}}_{i}$ is the predicted value of the i^th^ data and ${y}_{i}$ is its corresponding true value, for the total number of *n* data, the R^2^-score is defined as:

7 $${R}^{2}\left(y,\widehat{y}\right)=(1-\frac{{\sum }_{i=1}^{n}{\left({y}_{i}-{\widehat{y}}_{i}\right)}^{2}}{{\sum }_{i=1}^{n}{\left({y}_{i}-\overline{y }\right)}^{2}})\times 100,$$

in which $\overline{y }=\frac{1}{n}\sum_{i=1}^{n}{y}_{i}$. A perfect model returns each ${\widehat{y}}_{i}$ equal to its corresponding ${y}_{i}$ which leads to the ${R}^{2}$-score of 100% while a total mismatch returns R^2^-score of 0.

The other examination definitions for testing the validity of the applied model are $MSE=\frac{{\sum }_{i=1}^{n}{\left({y}_{i}-{\widehat{y}}_{i}\right)}^{2}}{n}$, $RMSE=\sqrt{\frac{{\sum }_{i=1}^{n}{\left({y}_{i}-{\widehat{y}}_{i}\right)}^{2}}{n}}$, and $MAE=\frac{{\sum }_{i=1}^{n}\left|{y}_{i}-{\widehat{y}}_{i}\right|}{n}$.

### Machine learning of the defect modes’ wavelength

As discussed, the three structural parameters, *p*, *q*, and *r*, affect the defect mode wavelengths. Considering the dependent variable, *Y*, as the defect mode’s wavelength and taking the independent variables, ${X}_{i}$ s as the structural parameters, *p*, *q*, and *r*, the MLR formula of Eq. (6) can be rewritten as:

8 $${\uplambda }_{j}= {\theta }_{{0}_{j}}+{\theta }_{{1}_{j}} p+{\theta }_{{2}_{j}} q+{\theta }_{{3}_{j}} r \mathrm{j }= 1\mathrm{ or }3.$$

In Eq. (8), j = 2 is omitted due to the constancy of the second defect mode wavelength (λ_2_) with changing *p*, *q*, and *r*.

In the first step, to simplify the MLR modeling, we take the dependency of ${\uplambda }_{j}$ to only two parameters. we take once θ_1_, θ_2_, or θ_3_ in Eq. (8) equal to zero. This way three datasets are gathered that are named **rq**, **rp**, and **pq**. Extending our model to cover the dependency of the ${\uplambda }_{j}$ s to all structural parameters leads to a combined dataset named **pqr**. With sweeping over *r*, *q*, and *p* with the values of 1 to 10, the size of our **pqr** dataset reaches a maximum of 1000. The results of ${\uplambda }_{j}$ MLR modeling, their regression coefficients, intercepts, and R^2^-scores for each dataset are reported in Table 2.

**Table 2:** Intercept, coefficients, and R^2^ score for MLR modeling of defect mode wavelengths for different datasets **rq**, **rp**, **pq**, and **pqr.**

		θ_0_	θ_1_	θ_2_	θ_3_	R^2^-Score (%)
λ_1_	**rq**	578.94	0	2.4421	2.0475	92
	**rp**	593.56	0.0263	0	1.9454	89
	**pq**	593.99	− 0.0497	1.9858	0	89
	**pqr**	582.99	0.1019	1.8265	2.0548	93
λ_3_	**rq**	665.62	0	− 2.6903	− 2.4112	92
	**rp**	648.44	− 0.1190	0	− 2.1950	91
	**pq**	647.43	0.0295	− 2.1536	0	87
	**pqr**	660.31	− 0.1179	− 2.0641	− 2.3411	91

Represented R^2^-score results in Table 2 which are above 87%, tell us the acceptable accuracy of our MLR model for each dataset. To be more precise, the R^2^-scores of the **rq** datasets are the highest among the other datasets with two dependent variables (**pq** and **rp)** which is demonstrative of the higher importance of *r* and *q* compared with *p* parameter in our model. This result which is in accordance with our physical discussions in the previous section is accompanied by near zero θ_1_ values (*p* coefficient in Eq. (8)), which reveals that the *p* parameter does not have an impressive effect on defect mode wavelengths. It is worth mentioning that taking the effect of all three parameters *p*, *q*, and *r* in modeling both defect modes lead to high accuracy with R^2^-score values above 90%. To ensure the reliability of our MLR model, we checked five-fold cross-validation score values which divide all available data into five randomly distributed subgroups, and tested the model with all these fives. The final cross-validation score is calculated by averaging these five values. Among the studied datasets, the cross-validation score above 90% is reached in the case of the **pqr**. Together with this high cross-validation score value, the low MSE and MAE values of 0.03 and 0.09 that are obtained from the MLR model of **pqr** dataset show the reliability of this dataset to model the wavelength of the defect modes.

**Figure 5:**
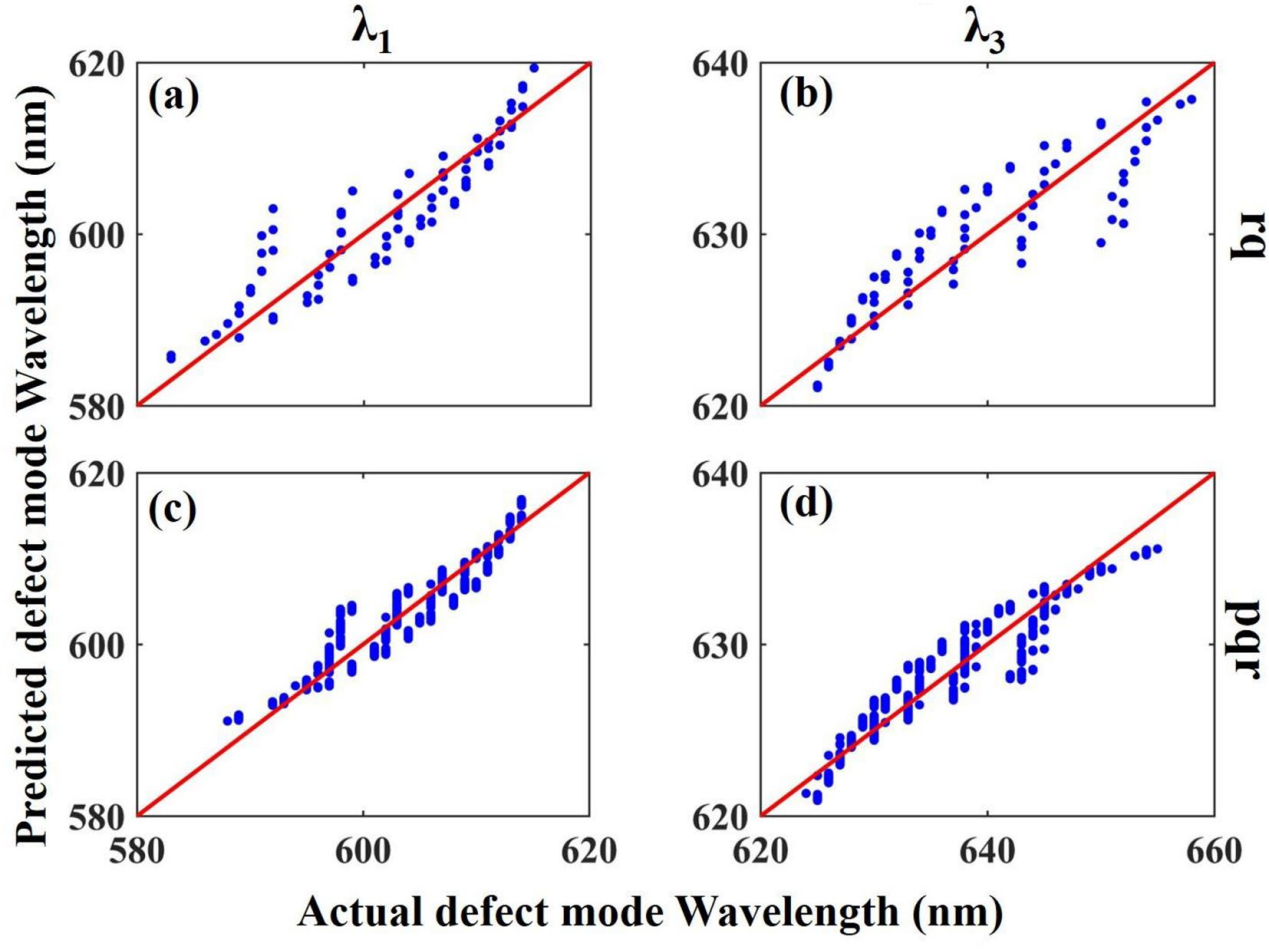
Predicted versus actual λ_1_ and λ_3_ for **rq** (**a**,**b**) and **pqr** (**c**,**d**) datasets. The redlines that are inserted in the plots demonstrate the equality of all actual and predicted data which are lines with a slope equal to one.

The results of modeling λ_1_ and λ_3_ by two datasets of **rq** and **pqr** are shown in Fig. 5.

Scatter plots of predicted versus actual values of λ_1_ and λ_3_ for **rq** (Fig. 5a,b) and **pqr** (Fig. 5c,d) are shown in Fig. 5. The redline that is inserted in the plots demonstrates the equality of all actual and predicted data which is a line with a slope equal to one.

The modeling of the **pqr** dataset by the MLR modeling results in the formulation of λ_1_ and λ_3_ as:

9 $${\lambda }_{1}=0.10p+1.82q+2.05r+582.99,$$

10 $${\lambda }_{3}=-0.11p-2.06q-2.34r+660.31.$$

According to the coefficients of the structural parameters in Eqs. (9) and (10), it can be concluded that both λ_1_ and λ_3_ mostly dependent on the change of *r* and *q* parameters rather than *p*. The positive/negative coefficients of all three parameters in Eqs. (9), (10) match well with the results of Fig. 4a,c regarding the redshift/blueshift of λ_1_/λ_3_ with increasing *r* and *q,* respectively.

### Machine learning of the defect modes’ absorption

Considering the absorption value of the defect modes (${A}_{k}$ s) as the dependent and structural parameters (*p*, *q*, and *r*) as the independent variables in the MLR modeling, Eq. (6) results in:

11 $${{\text{A}}}_{k}= {\theta {\prime}}_{{0}_{k}}+{\theta {\prime}}_{{1}_{k}}p+{\theta {\prime}}_{{2}_{k}}q+{\theta {\prime}}_{{3}_{k}}r k = 1, 2, 3.$$

Compared with the constancy of the second defect mode wavelength with changing the structural parameters, all three defect modes’ absorption values react to the change of *p*, *q*, and *r*. In the same procedure of modeling the defect mode wavelengths, in the first step, to simplify the model, we decrease the independent variables from three to two by using **rq**, **rp**, and **pq** datasets. Furthermore, by considering a dataset dependent on all three structural parameters, we investigate MLR modeling of ${A}_{k}$ s for four datasets of **rq**, **rp**, **pq**, and **pqr**. For each dataset, the value of the coefficients, intercepts, and R^2^-scores are reported in Table 3 for each defect mode.

**Table 3:** Intercept, coefficients, and R^2^ score for MLR modeling of defect modes’ absorption for different datasets **rq**, **rp**, **pq**, and **pqr.**

		θ′_0_	θ′_1_	θ′_2_	θ′_3_	R^2^Score (%)
A_1_	**rq**	0.6105	0	− 0.0548	0.0713	93
	**rp**	0.8087	− 0.0935	0	0.0424	96
	**pq**	1.2854	− 0.0882	− 0.0537	0	92
	**pqr**	1.1111	− 0.0971	− 0.0571	0.0490	93
A_2_	**rq**	0.6957	0	0.0805	− 0.0764	92
	**rp**	1.3593	− 0.1008	0	− 0.0590	92
	**pq**	0.8256	− 0.1068	0.0547	0	94
	**pqr**	1.1033	− 0.1095	0.0546	− 0.0504	93
A_3_	**rq**	0.5497	0	− 0.0360	0.0662	90
	**rp**	0.7574	− 0.0852	0	0.0472	95
	**pq**	1.2539	− 0.0793	− 0.0553	0	94
	**pqr**	1.0511	− 0.0920	− 0.0558	0.0600	93

**Figure 6:**
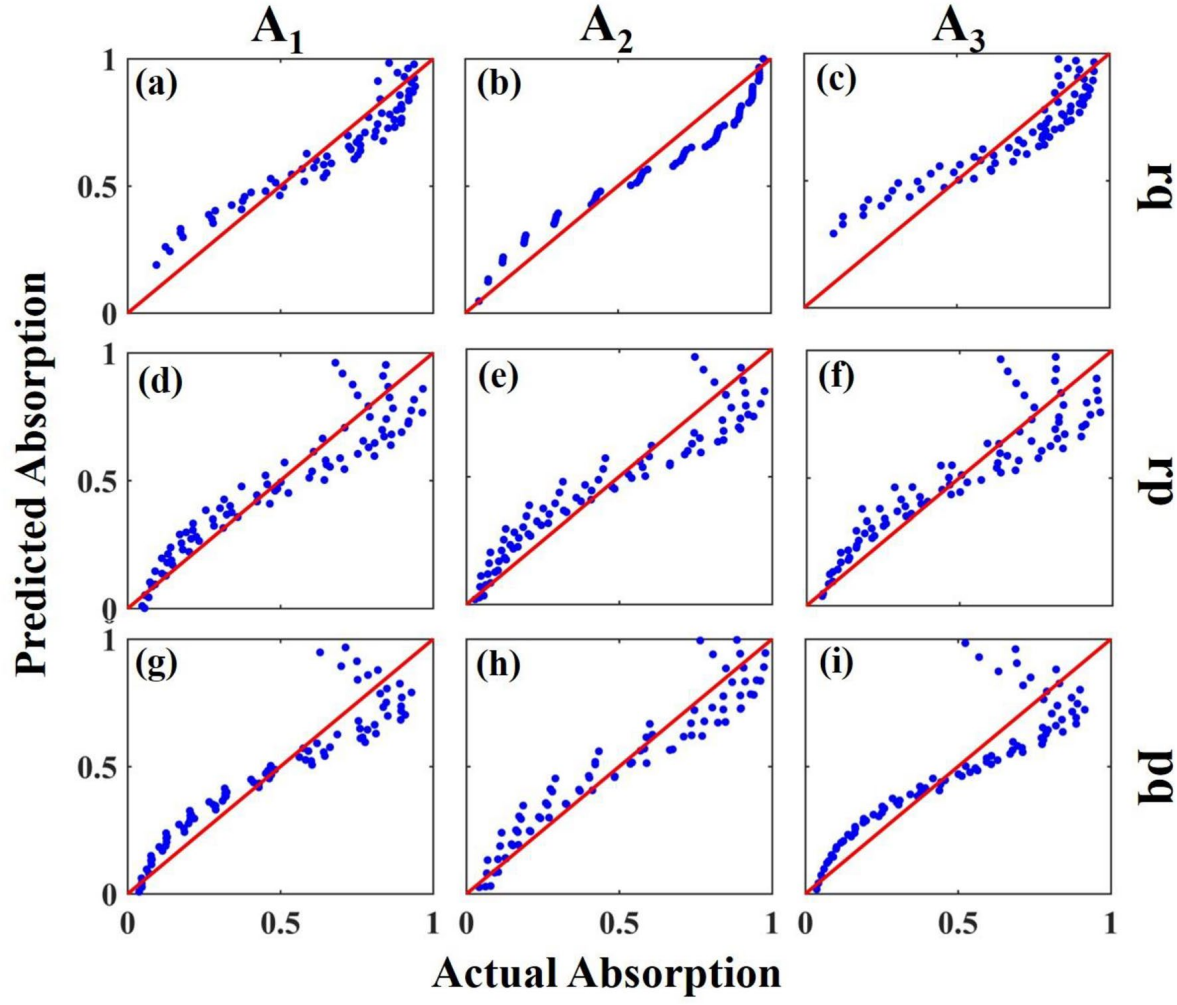
Predicted versus actual A_1_, A_2_, and A_3_ for **rq** (**a**–**c**), **rp** (**d**–**f**), and **pq** (**g**–**i**) datasets. The redlines that are inserted in the plots demonstrate the equality of all actual and predicted data which are lines with a slope equal to one.

The results of Table 3 show that the MLR modeling has an impressive performance in modeling the absorption of defect modes duo to the values of the R^2^-score, which are higher than 90% for all datasets. According to the obtained R^2^-score values, it can be concluded that all four datasets lead to acceptable predicted values. For example, the **rp** dataset has the best result for model A_1_ with an R^2^-score value of 96%, while the best result for modeling A_2_ is achieved using the **pq** dataset with an R^2^-score value of 94%. The results of MLR modeling for three defect modes’ absorption based on **rq**, **rp**, and **pq** datasets are demonstrated as scatter plots in Fig. 6a–(i).

The predicted values of A_1_, A_2_, and A_3_ versus the actual values for the **rq** dataset (first row of Fig. 6)/**rp** (second row of Fig. 6)/**pq** (third row of Fig. 6) are demonstrated in Fig. 6a–(i), respectively. The accuracy of our MLR model can be implied through the proximity of the scatters to the $y=x$ red line, which shows that the value of the predicted and actual data is close to each other.

**Figure 7:**
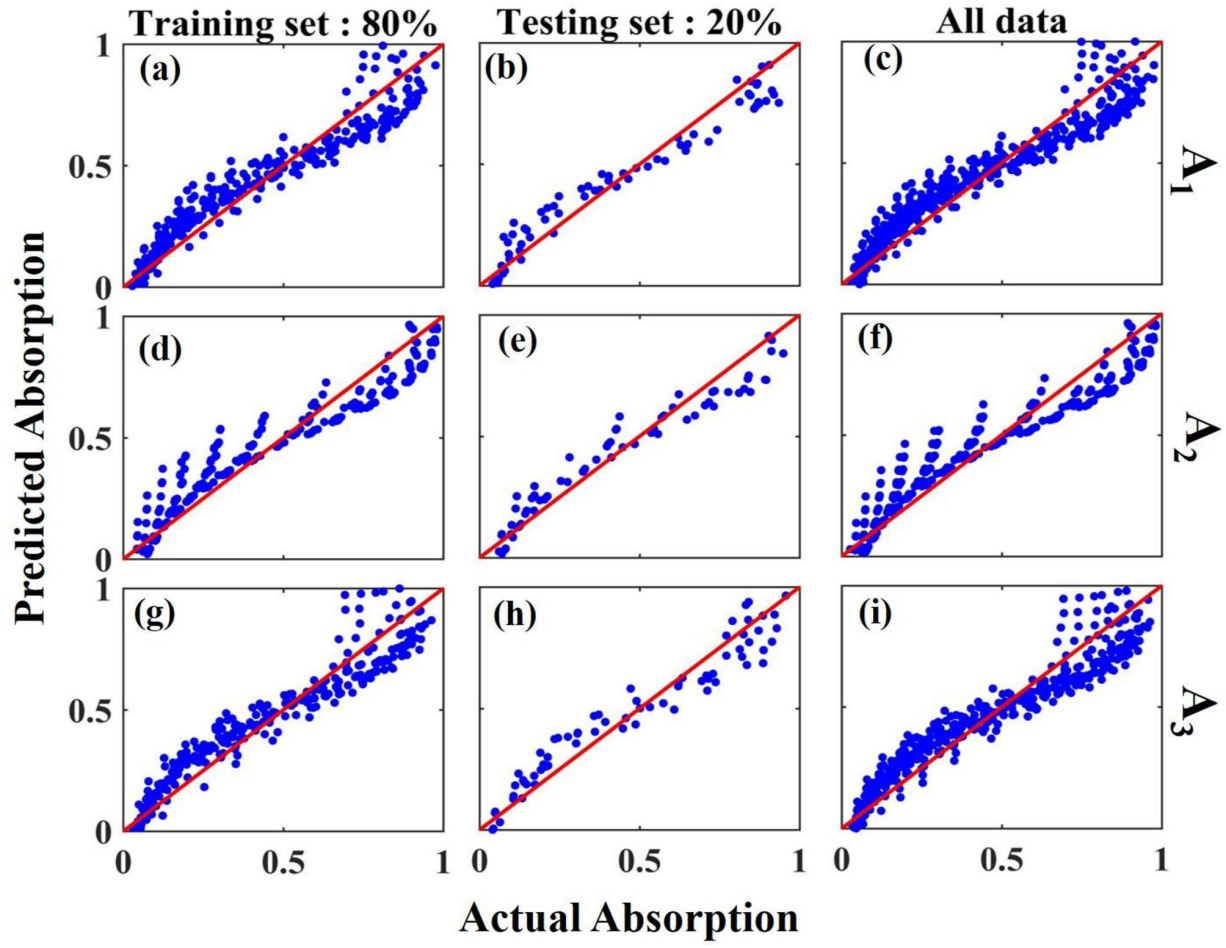
The predicted versus actual absorption for train, test, and all data of **pqr** dataset for A_1_ (**a**–**c**), A_2_ (**d**–**f**), and A_3_ (**g**–**i**). The redlines that are inserted in the plots demonstrate the equality of all actual and predicted data which are lines with a slope equal to one.

Separation of the **pqr** dataset to the training subset with 80% (the first column of Fig. 7) and the test subset with 20% (the second column of Fig. 7) of the data can be seen in Fig. 7a,b,d,e,g,h for studying A_1_/ A_2_/ A_3_. The third column of Fig. 7 represents the predicted versus actual data distribution considering all available data with the inclusion of the reference line, y = x, for A_1_/ A_2_/ A_3_ in Fig. 7c,f,(i).

By calculating the cross-validation score, MSE, and MAE for different datasets that are used to model the absorption value, we reached the best value of cross-validation score (above 90%), MSE (0.01), and MAE (0.07) values for the **pqr** dataset. In addition, the R^2^ score of this dataset shows an acceptable average value of 93% for all defect mode absorption modeling results.

According to the MLR modeling results, the absorption value of each defect mode based on the changes of three structural parameters *p*, *q*, and *r* is expressed in Eqs. (12), (13), and (14):

12 $${A}_{1}=-0.097p-0.057q+0.049r+1.11,$$

13 $${A}_{2}=-0.109p+0.054q-0.050r+1.10,$$

14 $${A}_{3}=-0.092p-0.055q+0.06r+1.05.$$

The machine learning technique used in this paper for predicting absorption value and wavelength of defect modes paves a new prominent way to avoid repeating the examination and simulation of the photonic devices with different structural parameters while the physics behind the excited modes remains unchanged.

## Conclusion

A symmetric DPC with three defects as DMD was proposed to achieve three narrowband defect modes with high absorption and wavelength adjustability in the PBG region (560 to 680 nm). Notably, absorption values of 92% and 93% for the first and third defect modes and 58% for the second defect mode with FWHMs less than or approximately equal to the remarkable value of 5 nm occurred at *p* = 4, *q* = 6, *r* = 8, and *t* = 8. The effect of changing the structural parameters (*p*, *q*, *r*, and *t* parameters) on the absorption and wavelength of defect modes was investigated. It was concluded that although all four structural parameters affect the absorption value of defect modes, only the distance between the defects (*r* and *q* parameters) adjust the wavelength of defect modes. An MLR modeling was implemented to assort the achieved data and predict the absorption value and wavelength of defect modes. The high R^2^-score and cross-validation score values (> 90%) confirm the perfection of MLR modeling for **pqr** dataset among all four datasets.

## Data Availability

The datasets analyzed during the current study is available and can be provided by the corresponding author upon a reasonable request.
